# A genetic algorithm to find optimal reading test word subsets for estimating full-scale IQ

**DOI:** 10.1371/journal.pone.0205754

**Published:** 2018-10-18

**Authors:** Ian van der Linde, Peter Bright

**Affiliations:** 1 Department of Computing & Technology, Anglia Ruskin University, Cambridge, United Kingdom; 2 Vision & Eye Research Unit (VERU), School of Medicine, Anglia Ruskin University, Cambridge, United Kingdom; 3 Department of Psychology, Anglia Ruskin University, Cambridge, United Kingdom; Istituto Di Ricerche Farmacologiche Mario Negri, ITALY

## Abstract

In clinical neuropsychology the cognitive abilities of neurological patients are commonly estimated using well-established paper-based tests. Typically, scores on some tests remain relatively well preserved, whilst others exhibit a significant and disproportionate decline. Scores on those tests that measure preserved cognitive functions (so-called ‘hold’ tests) may be used to estimate premorbid abilities, including scores in non-hold tests that would have been expected prior to the onset of cognitive impairment. Many hold tests entail word reading, with each word being graded as correctly or incorrectly pronounced. Inevitably, such tests are likely to contain words that provide little or no diagnostic power (i.e., can be eliminated without negatively affecting prediction accuracy). In this paper, a genetic algorithm is developed and demonstrated, using *n* = 92 neurologically healthy participants, to identify optimal word subsets from the National Adult Reading Test that minimize the mean error in predicting the most widely used clinical measure of IQ and cognitive ability, the Wechsler Adult Intelligence Scale Fourth Edition IQ. In addition to requiring only 17–20 of the original 50 words (suggesting that this test could be revised to be up to 66% shorter) and minimizing mean prediction error, the algorithm increases the proportion of the variance in the predicted variable explained in comparison to using all words (from *r*^2^ = 0.46 to *r*^2^ = 0.61). In a clinical setting this would improve estimates of premorbid cognitive function and, if an abbreviated revision to this test were to be adopted, reduce the arduousness of the test for patients. The proposed method is evaluated with jackknifing and leave one out cross validation. The general approach may be used to optimize the relationship between any two psychological tests by finding the question subset in one test that minimizes the prediction error in a second test by training the genetic algorithm using data collected from participants upon whom both tests have been administered. This approach may also be used to develop new predictive tests, since it provides a method to identify an optimal subset of a set of candidate questions (for which empirical data have been collected) that maximizes prediction accuracy and the proportion of variance in the predicted variable that can be explained.

## Introduction

A ‘hold test’ is a neuropsychological test that measures cognitive functions that remain relatively well preserved following neurological damage caused by traumatic brain injury, stroke, dementia or other condition. In longitudinal studies of preclinical to clinical populations, the relative preservation of hold test performance has been convincingly demonstrated [1]. Since, in neurologically healthy populations, performance in hold tests is highly correlated with that in non-hold tests [2], hold tests can be used with clinical populations to infer premorbid cognitive ability, such as full-scale IQ on the Wechsler Adult Intelligence Scale (WAIS-IV; [3]; for discussion see [4]). Knowledge of premorbid cognitive ability is essential both in evaluating the severity of impairment and in treatment planning.

Examples of hold tests that involve reading include the National Adult Reading Test (NART; [5–6]) and its international derivatives (which include NAART and AMNART [USA], [7–9]; NART-SWE [Sweden], [10]; NZART [New Zealand], [11,12]; fNART [France], [13]; DART [Netherlands], [14]; and AUSNART [Australia], [15], the Wechsler Test of Adult Reading (WTAR; [16]), the Test of Premorbid Functioning (TOPF; [17]), and a component of the Wide Range Achievement Test (WRAT4; [18]). Although the TOPF is intended to supersede the WTAR, the WTAR is still widely used and the NART also remains popular [19–21], particularly for research purposes.

To develop new neuropsychological tests, and to explore the relationships between those already in use, data from multiple tests are collected from healthy participants. In this way, the ability of hold-tests to predict the most likely results in other tests (such as full-scale IQ) can be evaluated (although subsequent longitudinal validation with preclinical to clinical populations is also desirable). In existing studies, a linear regression equation relating reading test performance to full-scale IQ is typically calculated (e.g., [12,20,21]. Ideally, a hold test would yield a perfectly linear correlation with a non-hold test of interest (*r* = ±1) and produce perfectly accurate predictions; however, in practice, this goal is unrealistic due to inherent limitations in test reliability and the imperfectly linear relationship expected between any two empirical datasets, especially when they measure different (albeit highly correlated) cognitive functions. The wealth of expertise and normative data relating to existing reading tests means that modifications either to the test or its corresponding instructions are undesirable without compelling justification. However, it is possible to use optimization and artificial intelligence (AI) techniques to develop new tests or revisions to existing tests that are demonstrably superior, or to identify more effective scoring procedures that may be applied to standard tests, e.g., by using question weighting schemes or question subsets that minimize the error between prediction and measurement with experimental data collected from participants upon whom both tests have been administered. In one recent study [22], a genetic algorithm (GA; [23]) was used to produce an abbreviated form of the Psychopathic Personality Inventory–Revised (PPI-R). In another, a GA was used to abbreviate the Multidimensional Experiential Avoidance Questionnaire [24]. Similarly, a GA with logistic regression to select the optimum combination of neuropsychological test results to predict progression to Alzheimer’s disease [25]. In the present study, rather than using genetic algorithms to abbreviate a test for comparison against results obtained using the full test, we use a GA to identify the optimum question subset from one test to most accurately estimate the result of a second (predicted) test.

A related area of research abbreviates tests on a per-participant basis. In Computerized-adaptive Testing (CAT) questions are selected based upon an estimate of current performance, and can yield accuracy comparable to an equivalent full-length test in which all questions are used [26]. In Multi-stage Testing (MST; [27]) a broadly similar approach is taken, except that banks of questions (so-called *testlets*) are selected at each decision stage. Using these approaches, sequences of decisions are made on-the-fly concerning which questions to present. However, such approaches are not appropriate in this case, where a core subset of questions is to be developed from which a single linear regression equation is desired, for which tests are administered by the clinician on paper (rather than using a computer). Additionally, the standardized instructions for the NART, used in this article to illustrate the general approach, require all items to be attempted for scoring to be valid. Furthermore, the approach presented is well suited to test design, enabling the researcher to develop new tests by establishing optimum combinations of questions that maximize predictive accuracy, potentially based upon a parent test (such as the NART).

To illustrate the general approach, data from the British NART [5,6] is used, in part because a recent survey indicates that is the most widely cited [21], but also because has been made freely available for use without restriction. It comprises 50 visually presented words that have irregular non-phonetic spellings and for which verbal responses elicited from participants are subject (by the experimenter, following standardized instructions) to binary classification as either having been correctly or incorrectly pronounced. The NART is scored by counting the number of incorrectly pronounced words (hereafter referred to as NART errors), and the instructions require that participants attempt all words for the scoring to be valid. The irregular nature of the words (i.e., their violation of typical phoneme-grapheme correspondence rules) is such that participants should be unable to spontaneously deduce correct pronunciations, and as such the test measures prior knowledge [28]. The set of 50 words that feature in the NART generally increase in difficulty through the test (thus the order that the words are presented is fixed, with words presented towards the end of the test intended to be less familiar to the target population). A patient who has suffered neurological impairment may therefore find the test rather onerous, particularly towards the end when presented with a sequence of increasingly difficult words. Furthermore, the intentionally ramped difficulty may disproportionately affect particular patient types for whom increased fatigue and impairments in concentration are apparent, making the use of an abbreviated test both faster to administer and less susceptible to confounds arising from patient fatigue.

At present, to predict premorbid intelligence using the NART, a linear regression equation is calculated in which the explanatory variable is NART errors and the predicted variable is, in the most recent standardization [20], WAIS-IV Full-scale IQ (FSIQ). A negative correlation (*r* < 0) is expected, such that an increase in the number of NART errors should yield commensurate reduction in predicted WAIS-IV FSIQ. In this paper, a GA is presented that increases the association between the NART and WAIS-IV FSIQ, reduces mean absolute prediction error, and reduces the number of words that participants are asked to pronounce. This approach is assessed for stability and overfitting via jackknifing [29,30] and exhaustive leave-one out cross-validation [31].

In recognition of the possibility that some NART words may provide little or no diagnostic power, and acknowledging that reduced test duration is desirable, in a recent study by McGrory and colleagues [32], Mokken scaling [33–34] was used to produce a reduced (and thus faster to administer) 23-word version of the NART. Referred to as the mini-NART, it was found to account for a similar proportion of variance in FSIQ as the full NART (44.8% *vs*. 46.5%). In this article, a markedly different approach is used that has several empirical advantages over the use of the full NART or the mini-NART: 1. it accounts for a greater proportion of the variance in measured WAIS-IV FSIQ; 2. residuals between predicted and measured WAIS-IV FSIQ using the identified NART subset are verified to be less than or equal to those observed using the full NART; 3. The number of words that participants are asked to pronounce is reduced from 50 (or 23 for the mini-NART) to 17–20 (around two thirds of the full test), suggesting that the test could be shortened, thereby reducing the likelihood of unnecessary fatigue. Furthermore, the method proposed simply requires the exclusion of individual NART words and the application of a revised regression equation, and can therefore either be administered as an abbreviated test or be applied retrospectively to existing data by rescoring the identified subset of words. The technique can, more generally, be used in the design of new predictive tests to identify an optimal subset of a set of candidate questions that yields the greatest coefficient of determination and smallest mean residual in relation to the measure that the test is intended to predict.

## Initial model

### Participants

An opportunity sample of 100 neurologically healthy adults were recruited primarily from University campuses in Cambridge and London, local retail outlets, and via social media, of which eight participants failed to complete one or more tests and were excluded from all analyses. There were no missing data across the sample of 92 participants (mean age 40 years; range 18–70; *s*_*age*_16.78), of which 30 were male, on any of the tests reported here. All were British nationals, with English as the first language, and with normal/corrected-to-normal vision and hearing. Participants self-declared that they had no history of neurological or psychiatric disorder. Extensive training in the administration and scoring of all tests was provided to three research assistants over several days by PB (an experienced neuropsychologist), and the testing sessions were closely monitored and supervised to ensure full compliance with the standardized administration and scoring procedures. All participants were recruited and tested between 2013 and 2016, in a UK University setting. The procedure was approved by the University Ethics Panel, and was conducted in accordance with the tenets of the Declaration of Helsinki. All participants had normal/corrected-to-normal vision and hearing (self reported), and spoke English as their first language.

### Data collection

All participants completed the NART first and then all 10 core subtests from the WAIS-IV battery. All tests were administered following standard published instructions. Participants attended a single session of approx. 90 minutes, with breaks provided upon request.

### Analysis procedure and results

**Fig 1:**
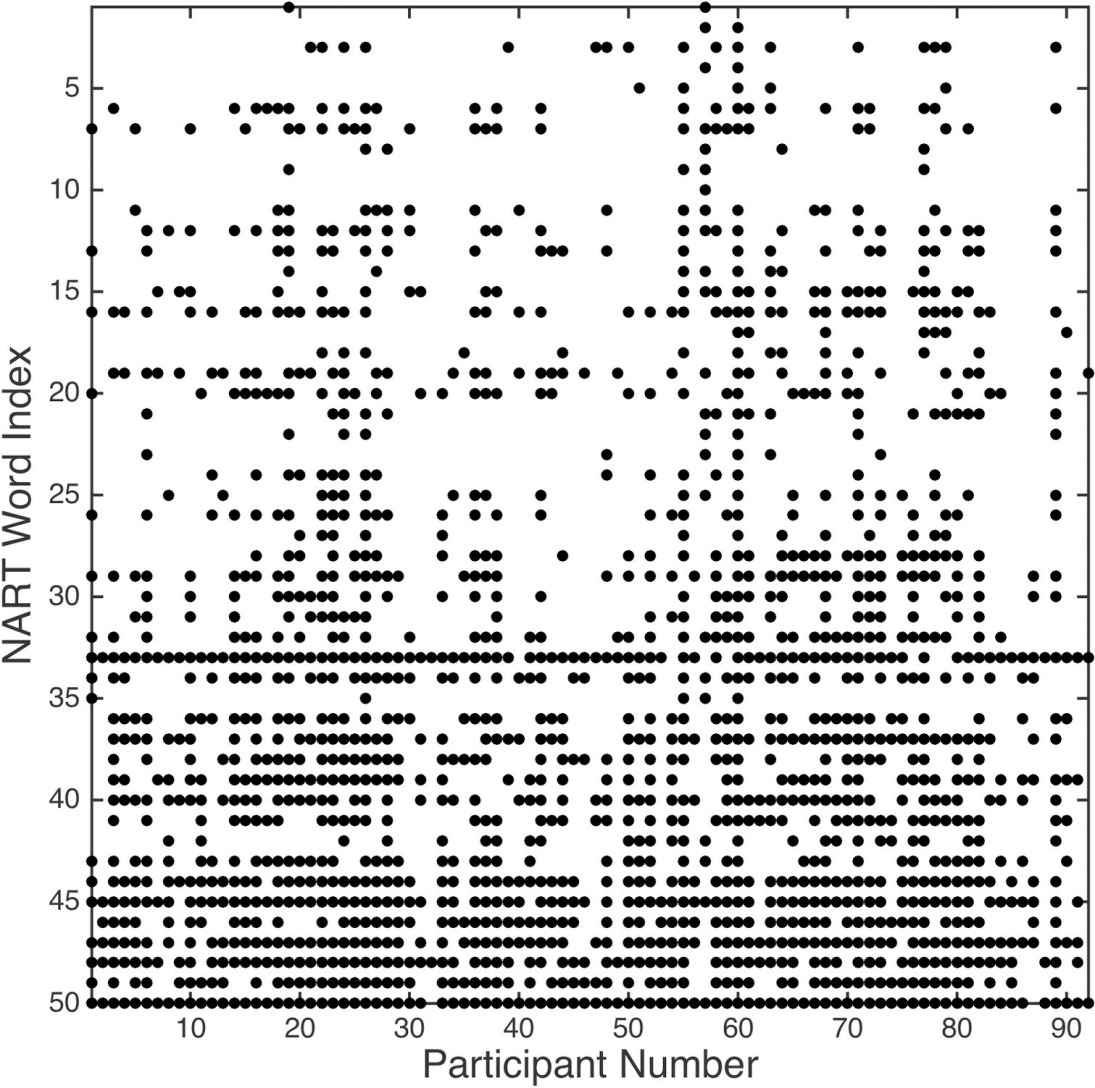
2-D bit matrix for all participants and NART word responses in which a black dot denotes a pronunciation error.

The NART responses for each participant were placed in a 2-D bit matrix, to be denoted *Q*, in which each row (1..*m*) corresponded to a NART word index, and each column (1..*n*) to a participant number (Fig 1). Here, rows *m* = 50 and columns *n* = 92.

The presence of a 1 in *Q* (a black dot in Fig 1) denotes an incorrect pronunciation (error), so the total number of NART errors, *x*_*j*_, for each participant *j* from 1..*n* over the sequence of NART words *i* from 1..*m*, is given by Eq 1, such that *x*_*j*_ ∈ [0..50]. The number of NART errors per participant in our data ranged from 2 to 46 ($\bar{x}=18.25,s_{x}=8.91$). Corresponding WAIS-IV FSIQ results, to be denoted *y*, ranged from 80 to 150 ($\bar{y}=108.52,s_{y}=12.71$). A Kolmogorov-Smirnov test indicates that neither empirical dataset deviates significantly from a normal distribution (*k* = 0.98, *k* = 1.00, both *p* < .0001).

$$x_{j}=\sum_{i=1}^{m}Q_{i,j},\;j=1,\ldots ,n$$ (1)

The linear Pearson product-moment correlation coefficient (PPMCC) between NART errors (*x*) and measured WAIS-IV FSIQ (*y*) is given by Eq 2. In addition to enabling a linear regression equation to be calculated (see below), the PPMCC, *r*, and coefficient of determination, *r*^2^, are commonly used in neuropsychological literature to assess the degree of association between different test scores (e.g., see [28]), and provide one metric against which the GA-derived model described later in this article is to be evaluated. The value given by Eq 2 for our data, consistent with that reported in [20], was *r*_(90)_ = −0.68 *p* < 0.000001, which is typically classified as *large* [35]. The coefficient of determination was *r*^2^ = 0.47, a comparable number to that reported in [5,6] and many subsequent studies that correlate NART error scores against earlier iterations of WAIS IQ. It suggests that the explanatory variable (NART errors) accounts for 47% of the variance in the predicted variable (WAIS-IV FSIQ).

$$r_{xy}=\frac{1}{ns_{x}s_{y}}\sum_{i=1}^{n}\left(x_{i}-\bar{x})(y_{i}-\bar{y}\right)$$ (2)

A linear regression equation (of the form $\hat{y}=ax+b$, where $\hat{y}$ denotes a predicted value of *y*) was produced, again in keeping with earlier approaches, with multiplicative constant *a* (slope) and additive constant *b* (*y*-intercept), which can then be used to predict WAIS-IV FSIQ ($\hat{y}$) for any number of NART errors (*x*). The PPMCC, *r* (Eq 2), is used to calculate the line equation constants (Eq 3 for slope, *a*, and then Eq 4 for *y*-intercept, *b*).

$$a=r\frac{s_{y}}{s_{x}}$$ (3)

$$b=\bar{y}-a\bar{x}$$ (4)

**Fig 2:**
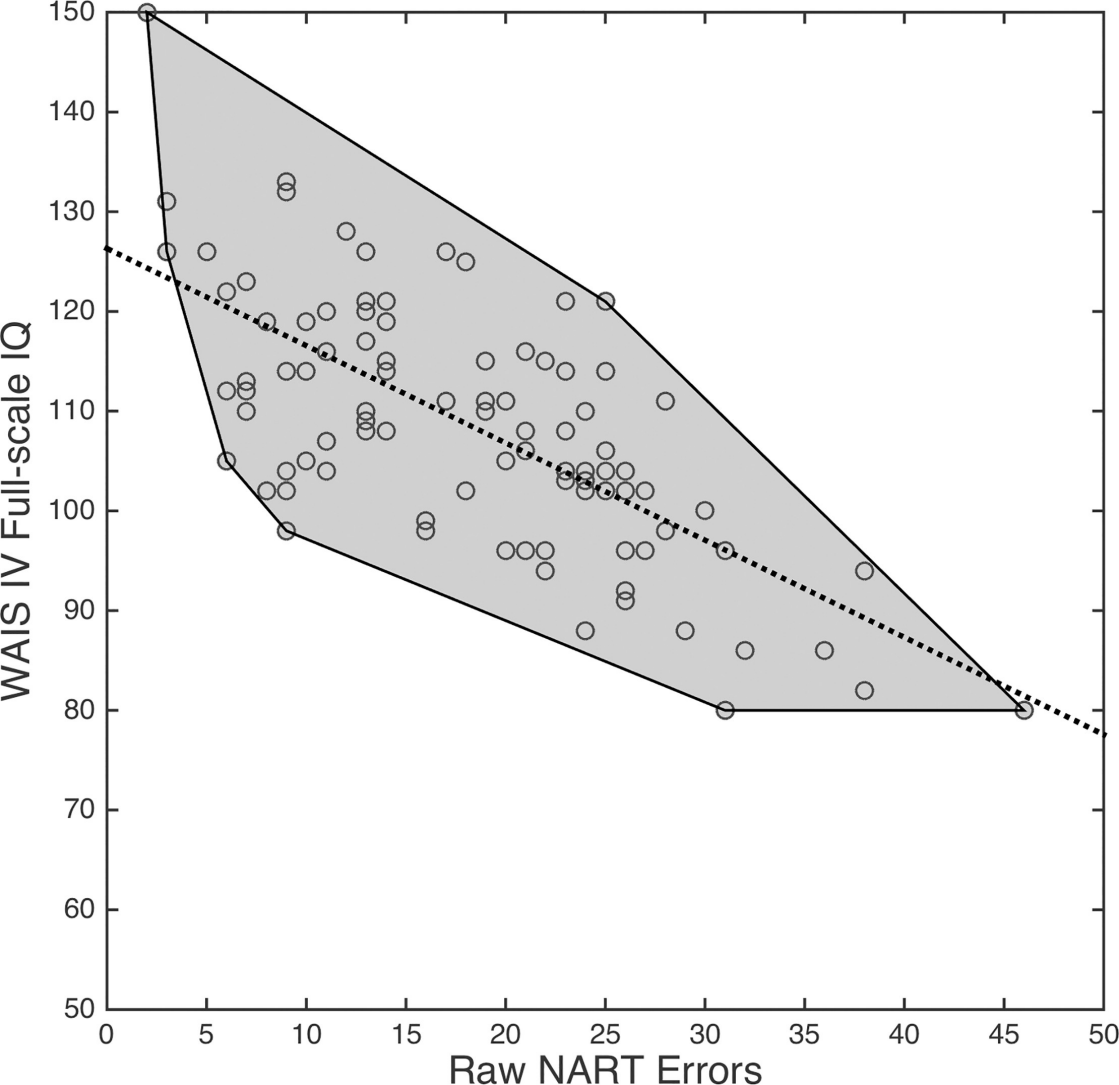
Scatterplot of raw NART errors *vs*. measured WAIS-IV FSIQ (hollow circles). Dotted black line is initial line equation; shaded zone is the convex hull.

Using the full set of NART words, the line equation for our data was $\hat{y}=-0.9750x+126.3163$, shown on a scatterplot of raw NART errors *vs*. WAIS-IV FSIQ in Fig 2 as a dotted black line with circles denoting measured values (i.e., our 92 participant test scores). The proximity of the sample points to the initial line equation is highlighted as a shaded zone (convex hull [36]).

A correlation coefficient (or coefficient of determination) should not, on its own, be used to assess the accuracy of a linear regression model such as this, since in a comparison between two hypothetical models, greater absolute *r* (or greater *r*^2^) for the first model may coincide with greater predictive accuracy for the second model, since the slope of a regression line is not necessarily coupled with lower average residuals (i.e., shorter average distance of measured sample points to their corresponding predictions). An additional metric should be used that specifically assesses the accuracy with which a model predicts known values; one simple metric that can accomplish this is mean absolute error (MAE, Eq 5), which has the advantage of being in the same units as the predicted variable (here, IQ points). Using raw NART errors, *MAE* = 7.33 (*s* = 5.64), showing that, on average, the error between predicted and observed WAIS-IV FSIQ using raw NART errors for our data was 7.33 IQ points.

$$MAE=\frac{1}{n}\sum_{j=1}^{n}\left|\hat{y_{j}}-y_{j}\right|$$ (5)

In addition, regression models should be validated to examine their stability to the removal of data points (i.e., the degree to which they may be affected by outliers), and their ability to make accurate predictions for samples not used in their production (i.e., the degree to which overfitting may have occurred). Alternative approaches to accomplish this include dividing data into training and testing sets, *k*-folds validation [37], and exhaustive leave-one-out cross-validation (LOOCV), described in [31]. The latter approach is used here, in part because it is fully reproducible (i.e., does not depend upon the randomized division of data into training and testing subsamples). In this form of validation, the predicted variable and other metrics of interest are calculated using models produced using subsamples of the original data in which one participant at a time has been left out (i.e., *n* subsamples of *n* − 1 participants, with participant *k* left out, such that *k* is iterated from 1.. *n*). These are sometimes referred to as jackknife samples. Thereafter, the accuracy with which each of the *n* models predict metrics of interest for the one left out participant not used their production is assessed. As before, *MAE* may be used to evaluate prediction accuracy both for the *n* jackknife models (which comprised *n*(*n* − 1) = 8372 individual predictions) and the *n* single left out sample predictions (here 92). A correlation coefficient (or coefficient of determination) can only be produced for the jackknife models since the left out samples are not associated with a single line equation.

For our data, averaging over the *n* jackknife models, with standard deviation shown in parenthesis, yields = −0.68 (0.01), *a* = −0.9750 (0.01), *b* = 126.3157 (0.28), and *MAE* = 7.33 (5.61). These values are remarkably close to where all participant data were used (reported above), indicating that outliers did not significantly affect these metrics. Next, the accuracy of the predicted variable elicited by each model using each respective single left-out participant (i.e., the participant not used in the production of that model) was assessed. This yielded *MAE* = 7.49 (*SD* = 5.78), which is fractionally greater than the *MAE* calculated using all data and the average *MAE* across the *n* jackknife models; however, this is to be expected given that the models are now being requested to make predictions for participants that were not used in their production. Furthermore, the differences in *MAE* values (between all data, 8372 jackknife subsamples, *n* leave-one-out samples) were not statistically significant (*p* > .05).

## Genetic algorithm model

### Apparatus

Statistical analyses and optimization algorithms were implemented in MATLAB (The Mathworks Inc., Natick MA). The standard regression mode, GA, validation routines and experimental data used for testing and validation are freely provided for download from the Open Science Framework (http://dx.doi.org/10.17605/OSF.IO/34BKU).

### Analysis procedure

The GA described below is charged with finding the optimum subset of NART words that yields the smallest average prediction residual (*MAE*), working from the initial starting point of using all 50 words.

GAs search solution spaces so large that they cannot feasibly be traversed using exhaustive/analytical approaches, enabling them to address computational problems, like the present one, that have no polynomial-time exhaustive solution. The final solution returned by a GA is not necessarily the best possible answer, since they rely upon an adaptive heuristic approach that iteratively improves upon each currently held solution until a solution that is deemed acceptably good is obtained. However, if appropriately configured, GAs can produce solutions that dramatically improve upon the initial starting point. GAs, being inspired by the biological principle of natural selection by survival of the fittest, entail the representation of candidate solutions as *chromosomes*, the evaluation of chromosome efficacy through a *fitness function*, the creation of new chromosomes via *mutation* and/or *crossover* (principally from the chromosomes identified as the most fit), and a *selection* method by which individual chromosomes are chosen to sire subsequent generations. A *termination* criterion must also be decided upon to determine how long the GA will run. Alternatives include letting the GA run for a fix period of time, for a fixed number of generations, until the solution is valid (e.g., in some NP-class problems in which finding a solution that merely works is a laudable goal), or until the fitness of the solutions produced over a pre-determined period of time or number of generations ceases to improve (i.e., evolutionary stagnation).

### Chromosome structure

Each chromosome, *c*, was a 1-D bit string (specifically, a sequence of 50 binary digits, each referred to as a gene) wherein each bit controls whether the NART word at index *i* should by used (*c*_*i*_ = 1) or not used (*c*_*i*_ = 0) in the calculation of each participant’s revised NART score. The number of alleles (alternatives) for each gene was therefore 2: 0 and 1. All possible solutions to the problem of finding the optimum NART subset can be represented on such a chromosome, of which there are 2^50^ (one quadrillion, one hundred twenty five trillion, eight hundred ninety nine billion, nine hundred six million, eight hundred forty two thousand, six hundred and twenty four), which is the cardinality of the powerset of the set of words (*w*) in the original NART, |℘(*w*)| = 2^|*w*|^ (i.e., the size of the set of all possible subsets of *w*). If one were to iterate through these subsets one at a time, a tight bound algorithm of exponential time complexity ϴ(2^|*w*|^) would be required, which is computationally impractical. To put this into perspective, if each subset took 1 sec to evaluate, it would take 36 million years to sequentially test all subsets to identify the true (guaranteed) optimum.

### Settings

The GA was run for a fixed number of generations (128), which was found to be more than sufficient for fitness to reach a stable asymptote (i.e., after which no further improvement in fitness was observed), and also provided an acceptable run-time of approx. 1–2 minutes on a standard computer. The number of children produced in each generation was also set to 128. With these settings, one run produces a total of 128 × 128 = 16384 candidate solutions, with a tendency to improvement from generation-to-generation that inevitably slows as the algorithm progresses and fitter solutions become more difficult to find. A mutation rate [38] of $\frac{5}{50}$, i.e. 10%, was used, determined experimentally to, when coupled with the use of 128 children per generation, yield fast and stable evolutionary descent.

The mutation routine entailed the negation of randomly selected genes (sometimes called *bit mutation* or *bit flipping*). Crossover (recombination) was not used, since this is not thought to be an effective approach for problems in which large changes in chromosome composition are likely to dramatically affect performance and thereby thwart evolutionary progress. A simple maximally elitist GA was used, such that only the fittest child in each generation was retained (determined as described below), which was set to be the parent of the subsequent generation. Other (less fit) chromosomes were destroyed. However, the fittest child in each generation always replaced the parent chromosome (whether fitter or not), enabling the fitness profile over time to decrease as well as increase, which is thought to prevent premature convergence (although it is acknowledged that the single-parent approach could lead to convergence to local optima, to demonstrate the general procedure, this simple approach was taken, and suitably fit solutions were indeed produced).

### Fitness function

To calculate chromosome fitness (a so-called *figure of merit*), first a revised NART response matrix, *Q*′, is calculated from *Q*, the original response matrix, by multiplying each participant’s NART word responses with the chromosome to be evaluated (Eq 6). This had the effect of masking the responses for specific words so that they no longer contributed to the final score for any participant.

$${Q\prime }_{i,j}=\sum_{i=1}^{m}Q_{i,j}\cdot c_{i},\;j=1..n$$ (6)

Next, the number of NART errors for the surviving NART words only, to be called the revised NART score, *x*′, is calculated using Eq 1 with *Q*′ substituting for *Q*. Next, a correlation coefficient is calculated using Eq 2 with *x*′ substituting for *x*, and then revised line equation constants, *a*′ and *b*’, are calculated using Eqs 3 and 4 with $\bar{x\prime }$ and *s*_*x*′_ substituting for $\bar{x}$ and *s*_*x*_, respectively. A revised prediction, $\hat{y\prime }$ can then be calculated for each participant. Using Eq 5, with $\hat{y_{j}^{\prime }}$ substituting for $\hat{y_{j}}$, the *MAE* using the adjusted NART scores can then be calculated, evaluating how accurately the current word subset approximates measured WAIS-IV FSIQ. The *MAE* value is returned as the fitness of the chromosome.

Over successive generations, the GA identifies the optimum subset: i.e., the optimum values in *c*, that when entrywise multiplied with the raw NART responses from all participants identically, minimizes *MAE*. As a consequence of falling *MAE*, the absolute correlation and coefficient of determination will also typically increase from generation-to-generation. It is worth noting that there may be multiple equally fit chromosomes, and that running the GA on multiple occasions could produce subtly different word subsets each time because several words or word combinations may each be equally suitable alternatives in *c* for *MAE* minimization (reflecting the randomness inherent in evolutionary descent, akin to nature). Selecting for the additional criterion of minimal subset cardinality is one approach that could be used to select from among equally fit alternatives (i.e., when two subsets yielding equal *MAE* are evaluated, particularly if the objective was to devise an abbreviated test or a new test using the fewest number of candidate questions).

### Model results

**Fig 3:**
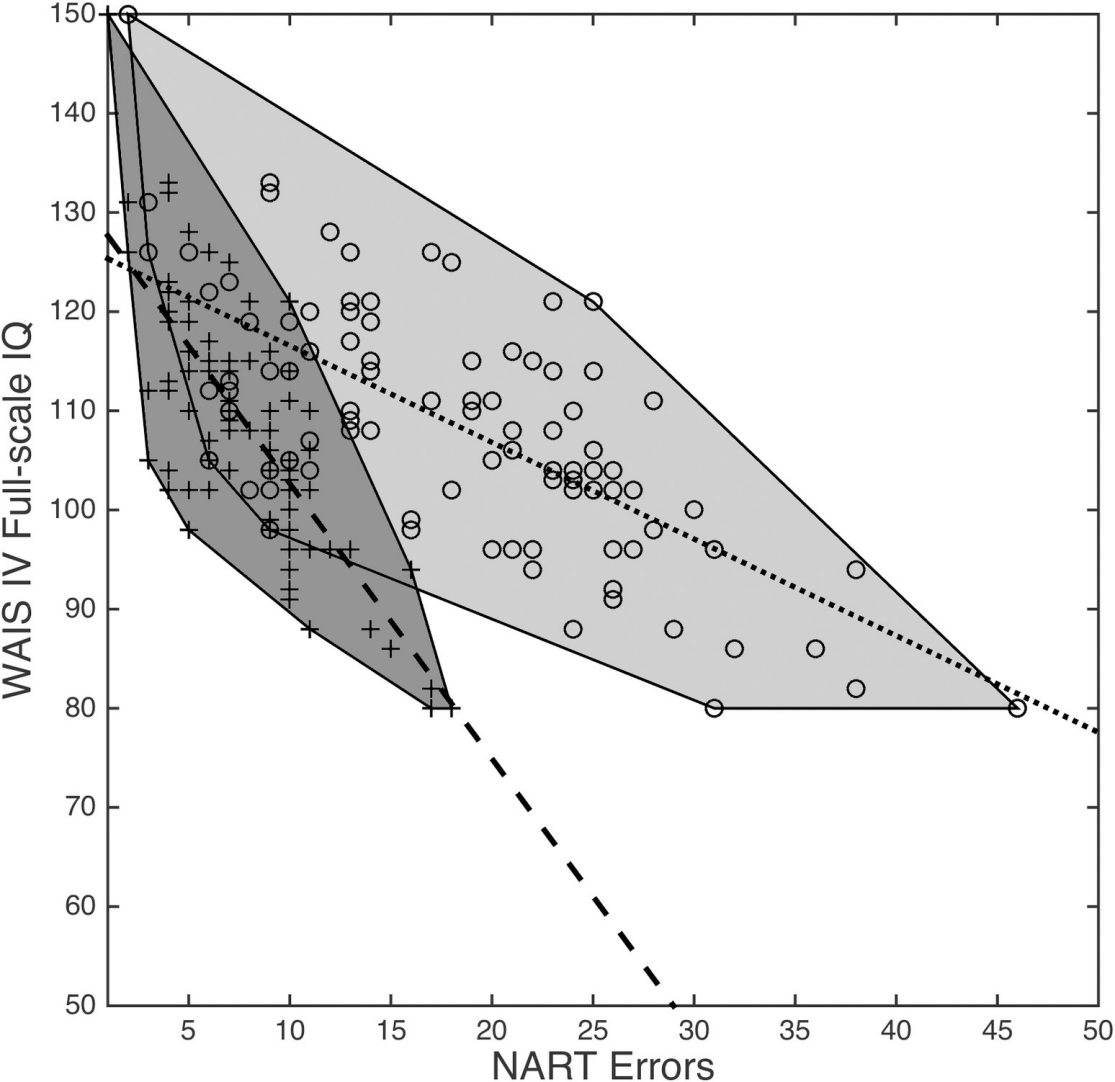
Scatterplot of NART errors *vs*. WAIS-IV FSIQ using original (sample points denoted ○, line equation dotted, light gray convex hull) and GA-derived (sample points denoted +, line equation dashed, dark gray convex hull) models.

Using the settings described above, repeating the GA for 100 runs, with standard deviation shown in parenthesis, yields *MAE* = 5.76 (0.03), *a* = −2.84 (0.16), *b* = 130.18 (1.50), *r* = −0.78 (0.003), and *r*^2^ = 0.61 (0.004), with the average number of words at 19.65. Over the 100 runs performed, 14 unique chromosomes were produced. Of these, the GA identified an optimal subset comprising 17 of the 50 original NART words. Despite using only around one third (35%) of the words in the full NART, this subset produces *more* accurate WAIS-IV FSIQ predictions (*MAE* = 5.75 relative to 7.33), and also yields a PPMCC of *r* = −0.78 compared to *r* = −0.68 for the original approach (the corresponding coefficient of determination, *r*^2^, increased from 0.46 to 0.61). The line equation constants for this chromosome were *a* = −3.4882 and *b* = 132.7113. The NART word subset identified by the GA on this run (with original NART word index shown in parenthesis) was: Capon (7), Nausea (9), Courteous (11), Naïve (14), Thyme (17), Procreate (23), Gouge (25), Superfluous (26), Simile (27), Façade (31), Drachm (33), Idyll (38), Puerperal (39), Leviathan (43), Prelate (45), Demesne (47), and Campanile (50). Raw and GA-derived participant scores (shown as ○ and + symbols, respectively) and corresponding line equations functions (shown as solid and dotted lines) are shown in Fig 3.

**Fig 4:**
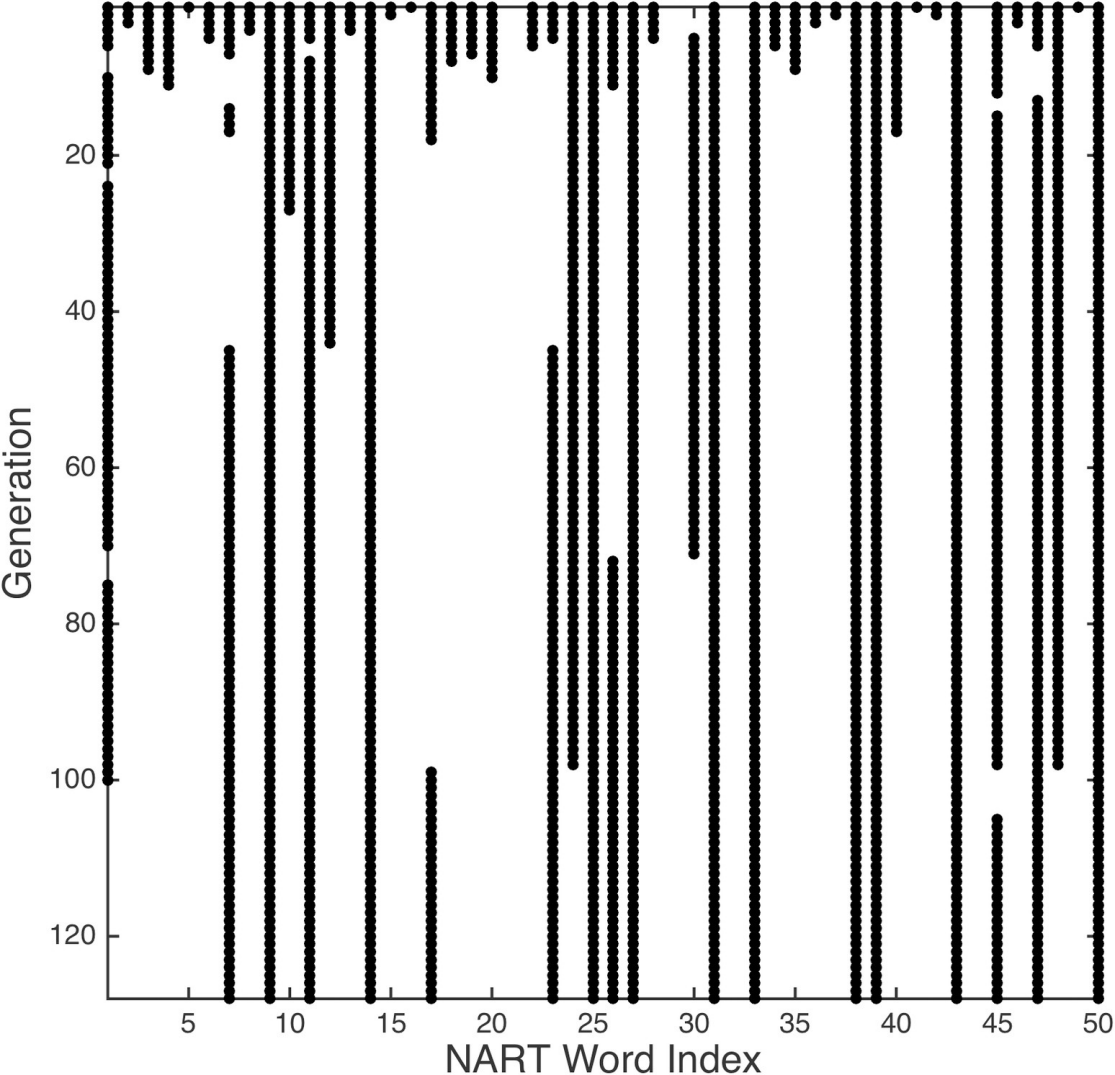
Chromosome evolution over 128 generations (top row is start point) in which black dots denote NART words used in the calculation of each participant’s revised NART score.

The evolutionary progression from the starting point of using all 50 NART words is shown in Fig 4, which also indicates that the number of generations that the GA was permitted to run was adequate for a stable chromosome to be formed using the mutation rate and number of children per generation described above.

### Validation

**Fig 5:**
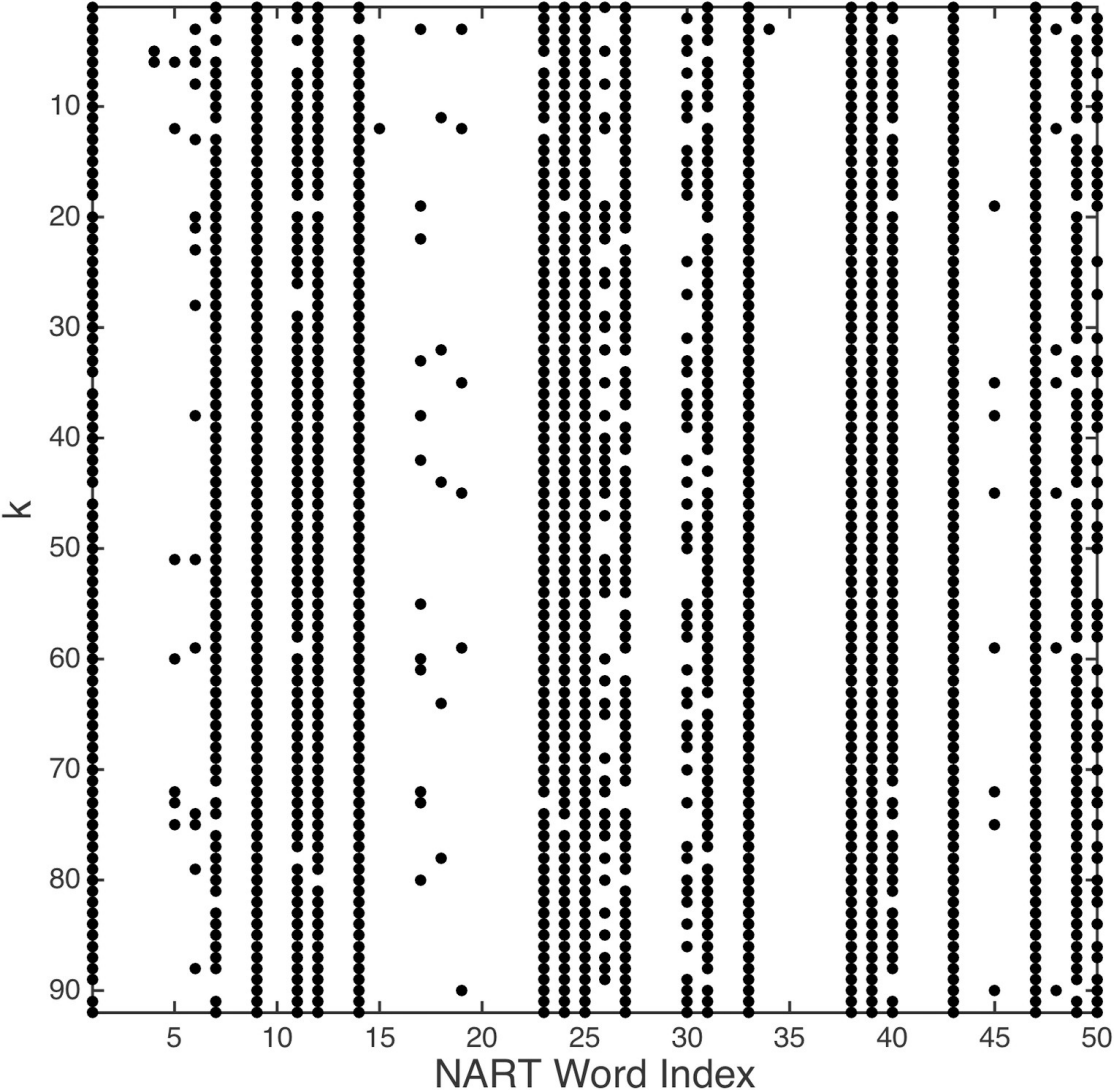
Chromosomes for *n* jackknifed GA models in which participant *k* was left out.

As with the initial regression model (above), the robustness and generalizability of the GA-derived model can be evaluated with jackknifing and LOOCV. For the GA-derived approach, averaging over the *n* jackknife models, with standard deviation shown in parenthesis, yields *r* = −0.78 (0.01), *a* = −2.8064 (0.14), *b* = 129.6049 (1.63), and *MAE* = 5.76 (5.45), values that are very close to the mean performance over 100 run using all participant data described above. The mean prediction was 108.69 (9.85). The chromosomes produced in each of these models are shown in Fig 5, in which a high degree of consistency implies a stable solution that is largely invariant to the removal of individual participants, and is quite consistent from run to run. For the *n* single left out samples, the *MAE* was 7.32 (6.11), with a mean prediction of 108.69 (9.79). The critical performance metrics (subset cardinality, mean prediction, *r*, and *r*^2^, and *MAE*) are shown in relation to the original model and its validation in Table 1. It is apparent that the cardinality of the subset (number of retained NART words) is fractionally higher than the ‘best’ runs using all data described above (in which 17 words are retained). Indeed, due to the heuristic nature of the approach, running the all-data single-run GA multiple times also produces some results wherein 19–20 words are retained, since these alternative solutions also yield an *MAE* = 5.75. It likely that, with if the number of participants were increased, *MAE* for single left-out participants would fall.

**Table 1:** Critical performance metrics for each models and cross validation set (standard deviation shown in parenthesis, were available).

	Initial Model	Cross Validation		GA Model (Best of 100)	GA Cross Validation	
		Jackknife	One Left Out		Jacknife	One Left Out
**Cardinality (Words)**	50	50	50	17	19.65 (0.80)	19.65 (0.80)
**Mean Prediction**	108.52 (12.71)	108.52 (8.64)	108.53 (8.66)	108.52 (9.94)	108.52 (9.85)	108.69 (9.79)
**PPMCC (r)**	-0.68	0.68 (0.01)	-	-0.78	-0.78 (0.01)	-
**COD (r**^**2**^**)**	0.46	0.46	-	0.61	0.61	-
**MAE**	7.33 (5.64)	7.33 (5.61)	7.49 (5.78)	5.75	5.76 (5.45)	7.32 (6.11)

In Table 1, it is apparent that the *MAE* for the all-data GA model and its jackknife subsamples are lower than the initial model all-data *MAE* and its jackknife subsamples. Comparing jackknife distributions using a *t*-test, this difference, although relatively small, is statistically significant [*t*_(8462)_ = 2.75,*p* < 0.01]. The *MAE* differences between the one-left-out (validation) sets in the initial and GA-derived models is not statistically significant (*p* = 0.84), despite that the GA-derived model uses, on average, only 19–20 words of the original 50, demonstrating that the additional words in the NART, as originally formulated, did not improve predictive accuracy for our data.

## Conclusions

A GA for optimizing the relationship between neuropsychological test data is presented and demonstrated using the NART and WAIS-IV FSIQ leading to increased absolute correlation/coefficient of determination, potentially reduced mean absolute error (i.e., smaller prediction residuals). The GA suggests that the number of words in the NART may be reduced by up to 66%; however, to evaluate the potential effects of reduced fatigue and alternate word order, the use of an optimal word subset should ultimately be evaluated by collecting data using it directly, rather than having data corresponding to the words identified as having predictive value being extracted from an administration of the full test. Due to the way that the GA was implemented, it could be that evolution to a local optimum occurred, and that a global optimum with higher performance is possible. It is also possible that incorporating other information, such as participant demographics and results on other hold tests (such as that WTAR and TOPF), may further elevate the correlation and reduce mean prediction error, rather like Johnson et al. [25] who used a GA to find the best combination of different neuropsychological tests to predict progression to Alzheimer’s disease (except that, here, questions from within tests would be selected, rather than tests themselves). Furthermore, greater performance might be achieved using artificial neural networks or other alternative AI approaches, or by weighting individual words rather than by the creation of optimal subsets. These possibilities are under investigation; however, the current article serves to illustrate a general principle that the strength of association between neuropsychological tests may be increased using GAs by, in this article, selecting optimum question subsets. A further caveat is that the cross-validation routines used here, although including LOOCV in which models are tested upon individual samples upon which they were not trained, may still be susceptible to a degree of overfitting; this possibility can be investigated in a follow-up study with a larger cohort which would support the division of participants into adequately sized training and validation sets.

It may also be the case that different locations (e.g., clinical centers, or geographic regions), participant demographics, and other clinical indicators may influence the optimal subset, so it may be more effective to select data to determine the optimal subset using one or more of these parameters, all without needing to adjust the basic NART test procedure, retaining the simplicity of administering this test for clinicians and enabling it to be used retrospectively on data already collected.
